# Fungal infections in pediatric patients with acute myeloid leukemia in a tertiary hospital

**DOI:** 10.3389/fpubh.2023.1056489

**Published:** 2023-03-22

**Authors:** Diana Ávila Montiel, Alberto Saucedo Campos, Martha Avilés Robles, Marco Antonio Murillo Maldonado, Rodolfo Jiménez Juárez, Marisol Silva Dirzo, Elisa Dorantes Acosta

**Affiliations:** ^1^Research Department, Children's Hospital of Mexico Federico Gómez, Mexico City, Mexico; ^2^Leukemia Cell Research Biobank, Children's Hospital of Mexico Federico Gómez, Mexico City, Mexico; ^3^Department of Infectious Diseases, Children's Hospital of Mexico Federico Gómez, Mexico City, Mexico; ^4^Department of Pediatric Hemato-Oncology, Children's Hospital of Mexico Federico Gómez, Mexico City, Mexico

**Keywords:** acute myeloid leukemia in children, invasive fungal diseases, Latin America, mortality, pediatric cancer

## Abstract

**Introduction:**

Acute leukemia accounts for more than 30% of all pediatric cancer cases, and of these, 15–20% are acute myeloid leukemia (AML). Children who super from AML are more likely to develop infections due to the humoral and cellular immune deficits generated by the disease and its treatment. The incidence of fungal infections is underestimated; reports show that up to 75% of fungal infections go undiagnosed until autopsy. In only 30 years, the incidence of invasive candidiasis has increased by 40-fold. Thus, the high morbidity and mortality associated with fungal infections in hematological patients make it necessary to adopt preventive measures.

**Methods:**

This work aimed to retrospectively identify pediatric patients with acute myeloid leukemia and invasive fungal diseases (IFDs) in a Latin American tertiary care hospital. A retrospective analysis of 36 clinical records of pediatric patients diagnosed with AML from 2007 to 2017 was carried out.

**Results:**

One hundred and twenty-nine hospitalizations were associated with infectious events. Thirteen patients in our study presented 15 infectious events associated with IFDs (11.6%). Two patients died because of complications related to IFDs (15.3%). The most frequent IFD type was aspergillosis, which was observed in 7 cases, followed by Candidemia, which was observed in 4 cases. The most frequent clinical manifestations were fever and respiratory distress.

**Discussion:**

Mortality due to IFD can be prevented with effective pharmacotherapy. An appropriate antifungal prophylaxis strategy still needs to be developed through larger prospective studies in Latin America.

## Introduction

Acute leukemia accounts for more than 30% of all pediatric cancer cases; of these, 15–20% are acute myeloid leukemia (AML) (1).

Children suffering from AML are more likely to develop infections due to the immune deficits and toxicity caused by chemotherapy.

Improvements in supportive care and the management of infections in neutropenic patients with early empirical antibiotic therapy have reduced mortality due to infectious disease from 15 to 20% in the late 1960's to <5% today (1).

Studies show that prophylaxis with antibiotics and antifungals reduces infection rates, hospitalization days, and mortality rates, but the emergence of resistance, particularly in vancomycin-resistant enterococci and gram-negative bacteria, is an emerging problem (2, 3).

Moreover, the frequency of fungal infections is underestimated, as shown by a study that analyzed the results of autopsies performed on patients with blood diseases; this study showed that 75% of fungal infections were not diagnosed while the patient was alive (4).

Due to the above, the 8th European Conference on Infections in Leukemia (ECIL-8) published guidelines for the use of antifungal prophylaxis in pediatric patients in 2021 (5).

Little is known about IFD-related mortality rates in Latin America. However, it is known that in this region, there is a higher incidence of AML (35.5% of all acute leukemia cases in pediatric patients) compared to that in Caucasian populations (5–13% of all acute leukemia cases in pediatric patients) (6–8).

Due to the higher incidence reported in Latin America, it is essential to update statistics and current information on the disease to improve treatment and develop health policies based on evidence (9–11).

Although various local efforts have been made to obtain epidemiological information in Mexico, it is still a work in progress.

In this work, we review the available information in a hospital specializing in childhood cancer in a developing country.

## Objective

The objective of this work was to retrospectively identify pediatric patients with acute myeloid leukemia and invasive fungal infections in a tertiary care institution in Latin America and to determine the clinical evolution and the stage of chemotherapy where these infections occurred most frequently.

## Materials and methods

This study was submitted/approved by the local research committee under the HIM-2019-046 register.

A retrospective analysis of clinical records of pediatric patients diagnosed with AML from 2007 to 2017 was carried out.

Patients from the study were treated with modified NOPHO AML-93 (12), except for one patient, who was diagnosed with AML M3 and treated with the protocol (IC-APL2006), based on the use of trans-retinoic acid and anthracyclines (13).

We collected sociodemographic variables, date of IFD, days of steroid administration, number of neutrophils at fungal infection diagnosis and duration of neutropenia, clinical course, site of fungal infection, and outcome.

Patients were classified according to the guidelines of the consensus definitions of the Infectious Diseases Group of the European Organization for Research and Treatment of Cancer and the Mycoses Study Group (2019) IFDs (14).

The selection of clinical records was carried out with a non-probabilistic sampling of consecutive patients. We collected available clinical records from patients who were treated from diagnosis until the outcome of death or surveillance at the Children's Hospital of Mexico.

## Results

Among 36 patients who had 129 hospitalizations secondary to infectious events, 15 invasive fungal diseases were identified in 13 patients (11.6% of all infectious events were from IFDs).

Of the 13 patients with IFDs (Table 1), six were females (46%), and seven were males (54%); regarding the AML diagnosis, the most frequent types of AML were AML M1 (*n* = 5) and AML M4 (*n* = 5). The most frequently observed translocations at diagnosis were Inv (16:16) (30.7%) in four patients, *t* (8:21) (23.2%) in three patients, *t* (15:17) (7.7%) in one patient, and *t* (9:11) (7.7%) in one patient. In four patients, molecular aberrations were not detected. The average age of onset of the disease was 9 years old, with a minimum of 1 year and a maximum of 18 years.

**Table 1:** Clinical characteristics in patients enrolled in this study.

**Patient**	**Age (years old)**	**Sex**	Molecular diagnosis^**^	**Morphological diagnosis according FAB**
1	16	Female	*t* (9:11)	AML M1
2	7	Male	*t* (8:21)	AML M2
3	1	Male	*t* (15:17)	AML M3
4	10	Male	Inv (16:16)	AML M4
5	1	Female	Inv (16:16)	AML M4
6	11	Male	NOT DETECTED	AML M1
7	16	Female	Inv (16:16)	AML M4eo
8	16	Male	NOT DETECTED	AML M4
9	15	Female	NOT DETECTED	AML M2
10	4	Male	*t* (8:21)	AML M2
11	14	Female	Inv (16:16)	AML M4eo
12	3	Male	NOT DETECTED	AML M1
13	2	Female	*t* (8:21)	AML M1

^**^The molecular diagnosis was performed with the Hemavision Kit, looking for the transcripts resulting from the most common chromosomal aberrations.

For 4/15 IFD events, an immediate history of prolonged steroid use was recorded (infectious events 1, 3, 4, and 6) (Table 2).

**Table 2:** Clinical course of IFDs in patients enrolled in this study.

**Infectious event**	**Patient number**	**Days since cancer diagnosis to IFD diagnosis **	**Steroid (days)**	**Steroid Indication and doses**	**Total neutrophils count at IFD diagnosis (× 10^6^/L) **	**Days with neutropenia (<0.5 × 10^9^/L) **	Working group according to reference (14)^**^	**IFD type according to insolation**	**Clinical data**	**Image diagnosis method**	**Treatment for IFD**	Antifungal prophylaxis^***^	**Site of infection **	Positive serology for galactomannans measured in optical units ^****^	**Clinical course after IFD**
1	1	26	83	Amphotericin B deoxycholate premedication hydrocortisone (1 mg/kg/dose IV)	23	20	Group 4	Chronic disseminated candidiasis	Fever	CT	Amphotericin B deoxycholate 5 mg/kg/dose every 24 h IV 14 days	No	Kidney and uterus	No	Clinical improvement
2	2	460	N/A	N/A	2,500	–	Group 3	Aspergillosis	Fever	CT	Amphotericin B deoxycholate 5 mg/kg/dose every 24 h IV 21 days	No	Lungs	Yes	Clinical improvement
3	3	44	10	Treatment of promyelocytic leukemia Dexamethasone 2.5 mg/m^2^ of body surface area every 12 h PO if WBC >5 x 10 9/L	280	60	Group 4	Candidemia	Fever	X-Ray of the thorax	Caspofungin loading dose 70 mg/m2 of body surface area, then 50 mg/m2 of body surface area dose, IV 14 days	Fluconazole	Systemic, blood	No	Death
4	3	52	15	Treatment of promyelocytic leukemia Dexamethasone 2.5 mg/m^2^ of body surface area every 12 h PO if WBC >5 x 10 9/L	150	60	Group 3	Aspergillosis	Fever, respiratory distress	CT	Amphotericin B deoxycholate 5 mg/kg/dose every 24 h IV 21 days	fluconazole	Lungs	No	Death
5	4	549	N/A	N/A	49	20	Group 9	Paecilomyces	Fever, respiratory distress	CT	Amphotericin B deoxycholate 5 mg/kg/dose every 24 h IV 28 days	fluconazole	Systemic	No	Death
6	5	40	15	Amphotericin B deoxycholate premedication hydrocortisone (1 mg/kg/dose IV)	40	10	Group 6	Aspergillosis	Fever	CT	Amphotericin B deoxycholate 5 mg/kg/dose every 24 h IV 21 days	Fluconazole	Lungs	Yes	Clinical improvement
7	6	74	N/A	N/A	40	10	Group 2	Agent not isolated	Fever	CT	Amphotericin B deoxycholate 5 mg/kg/dose every 24 h IV 14 days	Fluconazole	Unidentified focus	Yes	Clinical improvement
8	1	82	N/A	N/A	760	–	Group 3	Aspergillosis	Fever, respiratory distress	CT	Amphotericin B deoxycholate 5 mg/kg/dose every 24 h IV 14 days	No	Lungs	Yes	Clinical improvement
9	7	359	N/A	N/A	0	20	Group 4	Candidemia	Fever	CT	Caspofungin loading dose 70 mg/m2 of body surface area, then 50 mg/m2 of body surface area dose, IV 14 days	Nystatin	Systemic, blood	Yes	Clinical improvement
10	8	118	N/A	N/A	60	10	Group 3	Aspergillosis	Fever	CT	Amphotericin B deoxycholate 5 mg/kg/dose every 24 h IV 14 days	No	Lungs	Yes	Clinical improvement
11	9	117	N/A	N/A	0		Group 3	Disseminated Aspergillosis	Fever	CT	Amphotericin B deoxycholate 5 mg/kg/dose every 24 h IV 21 days	fluconazole	Systemic & Lungs	Yes	Clinical improvement
12	10	177	N/A	N/A	5	12	Group 3	Aspergillosis	Fever	CT	Amphotericin B deoxycholate 5 mg/kg/dose every 24 h IV 14 days	Nystatin	Lungs	Yes	Clinical improvement
13	11	68	N/A	N/A	10	10	Group 2	Agent not isolated	Fever	CT	Fluconazole 12 mg/kg/dose first day, then 6 mg/kg/day every 24 h PO 21 days	No	Unidentified focus	Yes	Clinical improvement
14	12	126	N/A	N/A	40	15	Group 2	Agent not isolated	Fever, respiratory distress	CT	Fluconazole 12 mg/kg/dose first day, then 6mg/kg/day every 24 h PO 21 days	No	Unidentified focus	Yes	Clinical improvement
15	13	79	N/A	N/A	350	10	Group 4	Chronic disseminated candidiasis	Fever	CT	Amphotericin B deoxycholate 5 mg/kg/dose every 24 h IV 14 days	Fluconazole	Cutaneous	Yes	Clinical improvement

^**^Working groups according to the consensus definitions of the Infectious Diseases Group of the European Organization for Research and Treatment of Cancer and the Mycoses Study Group (14).

^***^Fluconazole 6 mg/kg/day PO in tandem (3 times per week) until the patient reached a total neutrophil count of 500 and nystatin suspension was given topically at 100,000 units per milliliter *via* oral rinses 2 times daily.

CT, computed tomography; IFD, invasive fungal diseases; N/A, Does not apply.

^****^Positive serology for galactomannans measured in optical units (OU) through the sandwich ELISA method. Values above the cutoff point of 0.5 (OU) were considered positive.

For events 1 and 6, steroids were associated with premedication for patients who experienced infusion-related immediate reactions to amphotericin B deoxycholate (conventional). In those events, hydrocortisone (1 mg/kg/dose IV) was administered.

For events 3 and 4, steroids were related to treating the underlying oncological condition (AML M3). Dexamethasone was given at 2.5 mg per m^2^ of body surface area every 12 h PO if WBC >5 x 10^9^/L.

The mean number of total neutrophils at IFD diagnosis was 287, and the mean number of days of neutropenia was 21.

For 7/15 events, there was a history of receiving antifungal prophylaxis before IFD onset. For six events, fluconazole was administered at 6 mg/kg/day PO in tandem (3 times per week) until the patient achieved a total neutrophil count of 0.5 × 10^9^/L.

For two events (infectious events 9 and 12), nystatin suspension was given topically at 100,000 units per milliliter in oral rinses 2 times daily.

Positive serology for galactomannans was observed on 11/15.

The most frequent IFD type was aspergillosis in seven events, followed by candidemia in four cases. All patients presented with a fever, and in four patients, respiratory distress was observed.

Two patients died (patients three and four); the cause of death of both patients was directly attributed to IFD (one cause of death was pulmonary aspergillosis, and cause of death was secondary to systemic mycoses).

The treatment administered was in accordance with the guidelines of the Infectious Diseases Society of America (IDSA), however no consistency was observed between the prophylaxis administered, since fluconazole, nystatin, or no prophylaxis was found.

## Discussion

The toxic effects of chemotherapy, along with bacterial and fungal infections, are the main cause of morbidity and mortality in patients with AML and the reason AML patients have a lower survival rate (15–17).

The most relevant finding in this work concerns the high rate of IFDs in our patients, 11.6 vs. 6.9% as reported in the international literature (18).

In addition, the mortality rate was high (15.3%) in our hospital, despite being a tertiary-level hospital with personnel who specialize in treating these patients.

Routine antimicrobial prophylaxis (in some countries) and early identification of signs and symptoms related to sepsis in immunocompromised patients increase survival in children with AML (18, 19).

The diagnosis of invasive fungal diseases (IFDs), especially early diagnosis, is difficult because cultures have low sensitivity and fungi take several days to grow. The development of new rapid diagnostic techniques has improved the prognosis of these patients (20).

Since 2000, new drugs with proven antifungal activity have been developed, so the administration of broader primary prophylactic agents with maximum efficacy and minimum toxicity has been proposed.

Multiple studies show that broad-spectrum antibiotics, which are sometimes required but sometimes non-specific, are a highly relevant risk factor for developing systemic fungal infections. The incidence of invasive candidiasis has increased by 40-fold in 30 years, and certain yeast species that were previously considered commensals have demonstrated pathogenic capacity in certain circumstances. Something similar has happened with filamentous fungi. Thus, recent studies have shown that the incidence of invasive aspergillosis has increased 5-fold in the last decade (21, 22).

A multicenter study published by the cooperative group AML-BFM in 2004 included 405 pediatric patients; their research reported that 3% of patients were diagnosed with an invasive fungal infection, mostly during the remission induction phase (23).

Fisher et al. published a study with 871 patients from 38 children's hospitals; in this study, 57% of the cohort received fluconazole for antifungal prophylaxis. For patients who received antifungal prophylaxis, admission for infections was significantly less likely (3 vs. 6.9%; *p* = 0.007), with a statistically significantly decreased mortality risk (18).

The present work highlights the need to initiate prophylaxis protocols, especially given the frequency of aspergillosis in our population, since these events constituted 7/15 infectious events of the entire series of cases.

Significant risk factors associated with invasive aspergillosis have been described since their recognition constitutes the first step of prevention. Triazoles are the mainstay of antifungal prevention, while the other measures revolve around reducing exposure to mold spores (24, 25).

In the international literature, a consensus has been reached that additional data are needed regarding long-term prophylaxis to monitor adverse effects, the magnitude of the risk, if any, and the emergence of breakthrough invasive fungal infections (2).

A Canadian group published its guidelines for the use of antifungal prophylaxis in pediatric patients with acute myeloid leukemia and myelodysplastic syndrome. This group concluded that fluconazole is highly recommended during induced neutropenia for therapy (5).

Although the two patients who died received fluconazole prophylaxis, this work is important because it shows that IFDs cause death in children with AML in our population.

Leukemia and transplant centers should regularly monitor cases of invasive mold infection. An increase in incidence over baseline or the occurrence of invasive mold infections in patients who are not at high risk for such diseases should prompt evaluation for a hospital source.

Our study is limited regarding the number of patients; therefore, it is essential to characterize and describe our local epidemiology to make better clinical decisions and preventive actions.

## Conclusions

This retrospective study analyzed the medical records of children with AML who were treated in a tertiary hospital in Latin America.

Our patients had a high rate of infectious events; in addition, among the infectious events, 11.6% corresponded to IFDs, and this proportion exceeds that reported in the international literature.

Our patients generally had prolonged periods of neutropenia, which may lead to the occurrence of IFDs.

Unfortunately, no consistency was found in prophylaxis administration, and two patients died.

Knowledge of local epidemiology will allow disease management to begin with a solid basis and the implementation of prophylaxis protocols, as well as other measures that target the reduction of exposure to mold spores; these approaches could increase the overall survival rate of a series of patients in the medium term.

## Data availability statement

The original contributions presented in the study are included in the article/supplementary material, further inquiries can be directed to the corresponding author.

## Author contributions

DÁ and ED conceptualized and drafted the paper. AS drafted the paper and statistical analysis. MA, MM, and RJ drafted the paper. MS conducted fieldwork. All authors contributed to the article and approved the submitted version.
